# Poly(3-hydroxybutyrate)/Clay/Essential Oils Bionanocomposites Incorporating Biochar: Thermo-Mechanical and Antioxidant Properties

**DOI:** 10.3390/polym17091157

**Published:** 2025-04-24

**Authors:** Karla A. Garrido-Miranda, María Eugenia Gonzalez, Jacobo Hernandez-Montelongo, Andrés Jaramillo, Angelo Oñate, César Burgos-Díaz, Miguel Manso-Silvan

**Affiliations:** 1 Scientific and Technological Bioresource Nucleus (BIOREN-UFRO), Universidad de La Frontera, Temuco 4780000, Chile; 2 Center of Waste Management and Bioenergy-BIOREN, Universidad de La Frontera, Francisco Salazar 01145, Temuco 4780000, Chile; mariaeugenia.gonzalez@ufrontera.cl; 3 Departamento de Ciencias Matemáticas y Físicas, UC Temuco, Temuco 4813302, Chile; jacobo.hernandez@uct.cl; 4 Department of Mechanical Engineering, Universidad de La Frontera, Francisco Salazar 01145, Temuco 4780000, Chile; andresfelipe.jaramillo@ufrontera.cl; 5 Departamento de Ingeniería Mecánica, Universidad de Córdoba, Cr 6 #76-103, Montería 230002, Colombia; 6 Department of Materials Engineering (DIMAT), Faculty of Engineering, Universidad de Concepción, 315 Edmundo Larenas, Concepción 4070415, Chile; aonates@udec.cl; 7 Agriaquaculture Nutritional Genomic Center, CGNA, Temuco 4780000, Chile; cesar.burgos@cgna.cl; 8 Departamento de Física Aplicada and Instituto de Ciencia de Materiales Nicolás Cabrera, Universidad Autónoma de Madrid, 28049 Madrid, Spain; miguel.manso@uam.es

**Keywords:** antioxidant, biochar, bionanocomposites, PHB, essential oil (Tepa:eugenol)

## Abstract

The use of biodegradable active materials is being explored as a strategy to reduce food loss and waste. The aim is to extend the shelf life of food and to ensure biodegradation when these materials are discarded. The utilization of biodegradable polymers remains limited due to their inherent properties and cost-effectiveness. An alternative approach involves the fabrication of bionanocomposites, which offer a potential solution to address these challenges. Therefore, this study investigates the production of a polyhydroxybutyrate/biochar/clay/essential oil (Tepa:Eugenol) bionanocomposite with antioxidant and antimicrobial properties. The morphological, physicochemical, and antioxidant properties of the materials were evaluated in comparison to those of the original PHB. The materials obtained showed a porous surface with cavities, associated with the presence of biochar. It was also determined that it presented an intercalated–exfoliated morphology by XRD. Thermal properties showed minor improvements over those of PHB, indicating that the components did not substantially influence properties such as crystallization temperature, decomposition temperature, or degree of crystallinity; the melting temperature decreased up to 11%. In addition, the PHB/biochar_7/MMT-OM_3/EO_3 bionanocomposites showed a tendency toward hydrophobicity and the highest elastic modulus with respect to PHB. Finally, all essential-oil-loaded bionanocomposites exhibited excellent antioxidant properties against DPPH and ABTS radicals. The results highlight the potential of these bionanocomposites for the development of antioxidant active packaging.

## 1. Introduction

The Food and Agriculture Organization of the United Nations (FAO) has reported that, annually, 1.3 billion tons of food produced for human consumption are discarded or wasted around the world. This indicates that approximately one-third of global production is lost during the production, transportation, retail, and consumer stages or is disposed of in household waste [1]. Packaging has been identified as a key component in addressing the challenge of sustainable food consumption. It serves as the primary method for preserving food quality and extending shelf life [2,3]. In this context, active packaging is a type of packaging that interacts with food to extend its shelf life, improve quality and safety, maximize freshness, and minimize spoilage, resulting in a reduction of product waste. These packages can release antioxidant or antimicrobial compounds and act as oxygen and ethylene scavengers [4]. The principle of active packaging for ensuring food safety and quality lies in the interaction between the packaging material and the food. The active compounds contained in the material are released on the surface of the food (direct contact) or in the free space between the package and the food (indirect contact).

The active compounds based on essential oils exhibit excellent antioxidant, antibacterial, and antifungal properties. Essential oils are volatile, natural compounds, characterized by a strong odor, and are part of the flowers and leaves as a secondary metabolite [5]. In the food industry, essential oils have been highlighted for their antibacterial [6], antifungal [7], and antioxidant [8,9] properties. The most prevalent essential oils that exhibit optimal antioxidant and antimicrobial properties are carvacrol, thymol, and eugenol, all of which are extracted from oregano, cinnamon, and cloves, respectively [10]. However, a notable disadvantage of these essential oils is their distinctive aroma, which can negatively impact the sensory characteristics of food products. To mitigate this impact, essential oils can be blended with other oils that are less susceptible to altering the sensory profile of food items. In addition, blending oils can also result in a synergistic effect, where the combined properties of the blend may exceed the sum of the properties of each individual oil. This can enhance properties such as antimicrobial and antioxidant activity [11,12,13,14]. Consequently, various blends of essential oils have been studied to identify the most effective combinations [15,16]. Therefore, the present investigation proposes the mixture of Tepa oil (Laureliopsis philippiana [Looser] Schodde) and eugenol. The essential oil of Tepa is derived from the leaves and branches of a tree endemic to Chile, imparting a subtle forest aroma. Methyl eugenol, safrole, 3-carene, eucalyptol, and linalool have been described as the main components [17]. In addition, the antimicrobial properties of Tepa have been demonstrated against gram-positive and gram-negative bacteria [18,19]. Due to the properties of both oils, the Tepa:eugenol blend is a novel alternative as an active compound for active packaging.

The incorporation of active compounds into conventional packaging materials is a well-established practice. However, the current challenge lies in the development of biodegradable active packaging that can enhance the shelf life of food items (contributing to the reduction of food waste) and degrade under conditions conducive to composting, thereby mitigating the accumulation of plastic waste [20,21]. Researchers are undertaking extensive research on biodegradable plastics with the objective of establishing them as a viable alternative to conventional plastics utilized in food packaging. Polylactic acid, chitosan, polycaprolactone, starch, cellulose, and polyhydroxybutyrate [22,23,24,25,26] are among the most widely used biodegradable plastics. Polyhydroxybutyrate (PHB) is the most promising polyhydroxyalkanoate [27,28], since it has mechanical properties similar to those of conventional petroleum-based polymers. It is a semicrystalline polymer with a yellowish coloration. It has a high melting temperature, a high degree of crystallinity (60–80%), and a high glass transition temperature [29]. Additionally, it displays superior barrier properties (permeability to O_2_ or CO_2_) compared to low-density polyethylene [30]. However, it has some drawbacks, such as excessive brittleness and low thermal stability, which influence the melt blending process. Furthermore, PHB-based materials are susceptible to microbial attacks [31].

To increase the use of PHB as a biodegradable active material in food packaging, its mechanical and thermal properties need to be improved. An alternative to this problem is to obtain PHB bionanocomposites. These bionanocomposites consist of a matrix (consisting of biopolymers) and a filler (a nanofiller) and are regarded as a state-of-the-art method to improve the properties of biopolymers. Although PHB bionanocomposites can be obtained with different nanofillers (metal nanoparticles, nanocellulose, graphene, polymeric nanoparticles, cellulose nanofiber, among others) [32], nanoclays stand out for their natural character and high aspect ratio. Bionanocomposites of clay [33] exhibit superior performance to polymers or polymer blends in terms of barrier properties, mechanical strength, heat resistance, and optical properties [34,35] compared to traditional materials. The properties of these materials are determined by their morphology and the interactions between the polymer and the filler. This compatibility is a result of the ability to modify clays which allows for their dispersion in the polymeric matrix, obtaining an intercalated or exfoliated morphology [35,36,37]. PHB/clay bionanocomposites have demonstrated enhancements in mechanical properties, including Young’s modulus, tensile strength, and storage and loss moduli. Additionally, these composites have been shown to improve thermal stability and viscoelastic properties [38,39,40,41,42]. Specifically, the storage modulus has been shown to increase by up to 85%, as reported by Panayotidou et al. [22]. All these improvements are primarily achieved when an exfoliated morphology is present. An advantage of using clays to obtain an active material for food packaging is that it can act as a controlled release system for essential oils. As described by Mascheroni et al. [38], the release of carvacrol was controlled using 5 wt% clay and 15 wt% carvacrol, with an effect lasting up to 35 days. A similar study by Campos-Requena et al. [43] concluded that a higher amount of clay inhibits the release of carvacrol, resulting in a more prolonged effect over time.

Another alternative filler to PHB bionanocomposites is biochar. Biochar can be produced from forestry or industrial wastes through the process of pyrolysis or thermochemical conversion, in which biomass is heated to high temperatures (~>400–500 °C) under limited conditions or in the complete absence of oxygen [44,45,46]. The production of biochar derived from agricultural waste represents a sustainable approach to manufacturing this material. Some advantages that make biochar attractive as a filler material include its higher thermal stability, porous structure, high specific surface area, and low cost. The process of enhancing polymers with biochar can be executed through two primary methods: at the nanoscale or in composite form [47]. In one study on biochar with PHB by Haeldermans’ research group, who studied the effect of biochar on microstructure, crystallization, and thermal properties, it was found that biochar decreased the decomposition temperature while increasing the degree of crystallinity and the crystallization temperature of PHB for concentrations below 50 wt% biochar [48].

As described above, several studies have confirmed that the incorporation of clay or biochar improves the thermal and mechanical properties of PHB. In addition, essential oils have been reported to impart antimicrobial and antioxidant properties to both PHB [49] and PHB/clay composites [38]. However, to date there have been no reports of a material that combines PHB, biochar, clay, and essential oils in the same formulation, which could have application in active food packaging due to the properties they might present together. Therefore, the objective of this study is to develop new antioxidant materials with improved thermal and mechanical properties compared to PHB, using a mixture of PHB, biochar, clay, and essential oils through a solvent evaporation technique. The mechanical, physicochemical, and morphological properties of the bionanocomposite and PHB films are evaluated. Additionally, the antioxidant activities of the bionanocomposite films are assessed using DPPH and ABTS methods to evaluate their potential application in biodegradable active food packaging materials.

## 2. Materials and Methods

### 2.1. Materials

Poly(hydroxybutyrate) (PHB) was purchased from Biomer Ltd. (Krailling, Germany). The organic montmorillonite (MMT-OM) with the name Shelsite 30B Montmorillonite Nanoparticles (Nanoshell, Cheschire, UK), covered with quaternary ammonium groups, an APS less than 80 nm, purity of 99%, and density of 2.8 g/cm^3^, was used as nanoclay. Eugenol, natural (98%), was supplied by Sigma-Aldrich (Arlington Heights, IL, USA). The Tepa essential oil was provided by the research group of Dr. Jacobo Hernandez, extracted according to Perez-San Martin et al. [19]. Biochar derived from oat hulls was obtained according to the method described by González et al. [50] for which a pore diameter of 3.821 nm and a surface area of 4.077 m^2^/g was determined.

### 2.2. Preparation of Films of Bionanocomposites

**Figure 1:**
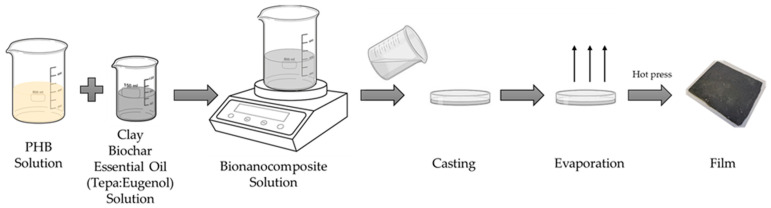
Scheme for the preparation of PHB/biochar/MMT-OM/EO_bionanocomposite films.

Initially, PHB was dissolved in chloroform at a ratio of 1:20, employing vigorous stirring at 60 °C for a duration of 3 h. Subsequently, the MMT-OM, biochar, and essential oil mixture Tepa:eugenol (70:30) (which will be referred to hereafter as EO) were dispersed in 10 mL of chloroform for 30 min using a sonicator. Thereafter, both solutions were amalgamated in a beaker with vigorous magnetic stirring for 30 min at 60 °C. The samples obtained are shown in Table 1. The milky solution was poured into a Petri dish and kept under hood for 12 h to remove the solvent by evaporation. Finally, the films obtained were pressed at 160° with a thickness of 1.00 ± 0.10 mm and were stored at 4 °C for further characterization. Figure 1 presents a schematic representation of the experimental procedures for preparing bionanocomposite films.

### 2.3. Physicochemical Characterizations

The surface morphology was examined by SEM using a JEOL electron microscope (Peabody, MA, USA), model JSM-6010, applying an acceleration voltage of 20 kV. PHB and the bionanocomposites were mounted in a holder and sputtercoated with gold. SEM array tomography was carried out in an electron beam lithography system (eLine Plus, Raith, Dortmund, NRW, Germany). The displayed image was captured at 5 kV with a 20 µm aperture, using the SE detector.

The surface wettability of bionanocomposites and PHB was evaluated with a water contact angle goniometer (KSV CAM-101, Scientific Instrument, Helsinki, Finland) used in the static sessile drop mode: a 3 μL drop of water was applied onto the sample surface and the contact angle formed with the surface was measured. Each measurement was repeated five times.

The chemical features of the samples were determined by Fourier transform infrared spectroscopy–attenuated total reflectance (FTIR–ATR, IRSpirit-X, Shimadzu, Kyoto, Japan) using direct transmittance. The samples were analyzed in the spectral region between 4000 cm^−1^ and 400 cm^−1^, with a resolution of 2 cm^−1^ and an average of 32 scan.

The crystalline and semicrystalline structures of the samples were examined using the Rigaku X-ray diffractometer Smartlab model, with a goniometer Theta–Theta Bragg-Brentano geometry and the solid-state detector D/teX Ultra 250 model (Rigaku Corporation, Tokyo, Japan). The diffraction patterns were recorded using Cu-Ka (λ = 1.5418 Å) radiation at 40 kV/30 mA. The measurement was achieved between 5 and 60° (2θ), with a step of 0.02° and a scanning speed of 1°·min^−1^.

The thermal profiles of the samples were measured using thermal gravimetric analysis (STA 6000, Perkin Elmer, Waltham, MA, USA). During the measurement, a weighted sample of about 6 mg was heated from 30 °C to 550 °C at a heating rate of 20 °C/min under a dry nitrogen atmosphere. The degradation temperature (Td) and the temperature after 5, 10, and 50% mass losses (T_5%_, T_10%_, and T_50%_) were calculated through the derivative of the TGA curves (DTG). The melting (Tm) and crystallization (Tc) temperatures, as well as the enthalpies of fusion (ΔHm) and crystallization (ΔHc) of the different bionanocomposites and PHB, were also determined by differential scanning calorimetry (DSC). The DSC was performed in a DSC Q100 (TA instrument, New Castle, DE, USA) in three stages, all at 10 °C/min: the first entailed heating from 25 to 200 °C, the second cooling from 200 °C to −20 °C, and the third heating from −2 0° to 200 °C. The degree of crystallinity (Xc) was calculated from the DSC curves as described by Garrido-Miranda et al. [51].

#### Nanoindentation

Nanohardness tests were conducted using a Hysitron TI 980 (Bruker, Minneapolis, MN, USA) apparatus. Test conditions were set with a 9 s loading cycle, characterized by a trapezoidal function comprising 3 s of loading, 3 s of dwelling, and 3 s of unloading. Measurements were performed via accelerated property mapping (XPM), maintaining a constant force of 100 µN and a data acquisition rate of 500 points per second, with a lateral movement speed of 0.5 µm/s.

The XPM test was executed with a 3 × 3 matrix of indentations, spaced 1 µm apart in both horizontal and vertical directions. A Berkovich diamond probe was utilized, possessing a Poisson’s ratio of 0.07 and a Young’s modulus of 1140 GPa.

To calibrate the probe, indentations were made in a H-pattern on a fused quartz sample, applying a load of 8000 µN. Subsequently, the probe was brought to the center of the specimen of interest until minimal contact was reached. The obtained results were adjusted using the probe’s area function relative to the depth of contact, as per the following equation:

(1) $$A_{c}\left(h_{c}\right)=24.5{h_{c}}^{2}$$

where $h_{c}$ represents the depth of contact and $A_{c}$ is the projected hardness impression area.

The hardness and elastic modulus were calculated from the recorded load–displacement curves (a method developed by Oliver and Pharr). The elastic modulus (E) was obtained from the initial slope of the load versus displacement curve according to Equations (1) and (2).

(2) $$\frac{1}{E_{r}}=\frac{1-v^{2}}{E}+\frac{1-v_{i}^{2}}{E_{i}}$$

(3) $$E_{r}=\frac{\sqrt{\pi }S_{max}}{2\sqrt{A}}$$

where $E_{r}$ is the reduced modulus, *E* is the elastic modulus, and *ν* and *ν_i_* are the Poisson proportions of the material; the corresponding values for the diamond tip are $E_{i}\;$(1140 GPa) y *ν_i_* (0.07) [52]. In Equation (2), S is the slope of the discharge curve dP/dh at the beginning of the discharge and A is the contact area between the material and the maximum load of the indenter.

On the other hand, the hardness (H) was determined, corresponding to the maximum point ($P_{max}$) of the load–displacement curve (Equation (4)).

(4) $$H=\frac{P_{max}}{A}$$

### 2.4. Antioxidant Activity via DPPH and ABTS

The antioxidant activities of the bionanocomposites and PHB were measured by analyzing the scavenging activity of the free radical DPPH (2,2-diphenyl-1-picrylhydrazyl radical), as previously reported by Garrido-Miranda et al. [38]. Measurements were performed by weighing 30.0 ± 2.0 mg of bionanocomposites or PHB into an amber vial, with 3 mL of DPPH solution (0.1 mM DPPH in methanol) added to the vials. A total of 100 µL of ascorbic acid (3 mM) was used as a positive control. The solution was left for 30 min at room temperature in the dark. Afterward, the absorbance (Abs) was measured with a UV-visible spectrophotometer (Evolution 60S, Thermo Scientific, Madison, WI, USA) at 515 nm.

The antioxidant activity of the samples was also analyzed by means of the radical cation ABTS (2,2′ azino-bis (3-ethylbenzothiazoline)-6-sulfonic acid), which was measured according to the method described by Kojom et al. [53], although with some modifications. Measurements were performed by weighing 30.0 ± 2.0 mg of bionanocomposites or PHB into an amber vial, with 3 mL of ABTS solution (with absorbance of 0.70 ± 0.20) added to the vials. A total of 100 µL of ascorbic acid (3 mM) was used as a positive control. The solution with the samples was incubated for 6 min and then measured at 734 nm.

The antioxidative activity (DPPH and ABTS) of all the samples was calculated as follows:

(5) $$\mathrm{Antioxidant}\;\mathrm{activity}\;(\%)=\left(\frac{{Abs}_{control}-{Abs}_{sample}}{{Abs}_{control}}\right)\times 100$$

where *Abs_control_* is the absorbance of the sample control (PHB) and *Abs_sample_* is the absorbance of the sample of bionanocomposites. The assay was carried out in triplicates.

## 3. Results and Discussion

**Figure 2:**
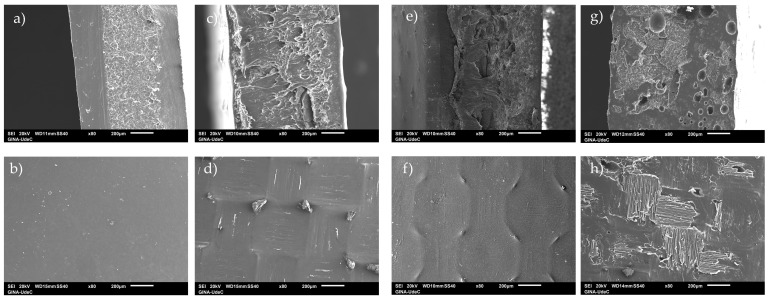
SEM images of PHB and different PHB/biochar/clay/EO bionanocomposites. (**a**,**b**) PHB; (**c**,**d**) PHB/biochar_3/MMT-OM_4/EO_3; (**e**,**f**) PHB/biochar_5/MMT-OM_4/EO_3; (**g**,**h**) PHB/biochar_7/MMT-OM_3/EO_3.

**Figure 3:**
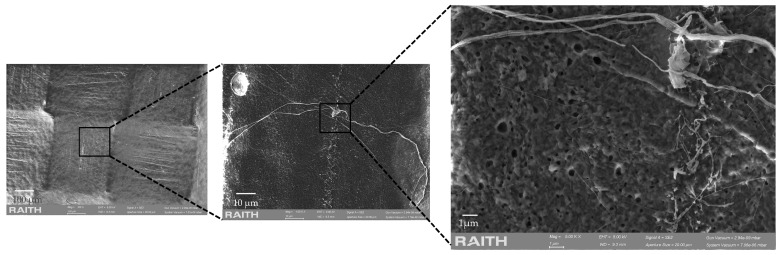
SEM images of PHB/biochar_3/MMT-OM_4/EO_3 bionanocomposites.

The surface of the samples was analyzed using scanning electron microscopy, and the results include SEM images of the surface and the cross-section of each film. It is important to know how the polymer interacts with the different fillers, as their interaction and, therefore, their morphology will influence the properties of the bionanocomposites. Figure 2a, corresponding to the polymeric matrix (PHB), shows a homogeneous smooth surface and the cross-section (Figure 2b) shows a rough zone and a homogeneous smooth zone. When looking at the bionanocomposite with the lowest concentration of biochar (Figure 2c,d), the surface shows some agglomerations, and the design of the Teflon used for pressing is observed. Increasing the concentration of biochar to 7% (Figure 2g,h) by weight resulted in the formation of a rough surface, which exhibited a pattern consistent with the Teflon used for pressing. The cross-section of the bionanocomposite films revealed a surface characterized by numerous small cavities, attributable to air bubbles, and lamellae (Figure 2h). The observed discrepancy in morphology can be attributed to the bionanocomposite loading material. Specifically, the clay functions as a reinforcing agent for the polymer, thereby resulting in the formation of flakes or lamellae [51]. Also, the effect of the presence of biochar, which is known to be a material with high porosity, on the bionanocomposite can be observed in Figure 2h. Authors such as Shiwang Liu et al. [54] have also observed that biochar presents trenches on the surface and internal cavities (tunnels). Figure 3 shows different SEM images of the surface of the PHB/biochar_3/MMT-OM_4/EO_3 bionanocomposite obtained by SEM, where it is observed that the bionanocomposite shows a highly porous surface, with pore sizes from 1 um to 0.5 μm. Other investigations have determined that the polymers are able to infiltrate the pores, resulting in a similar morphology to that of these bionanocomposites [55].

**Figure 4:**
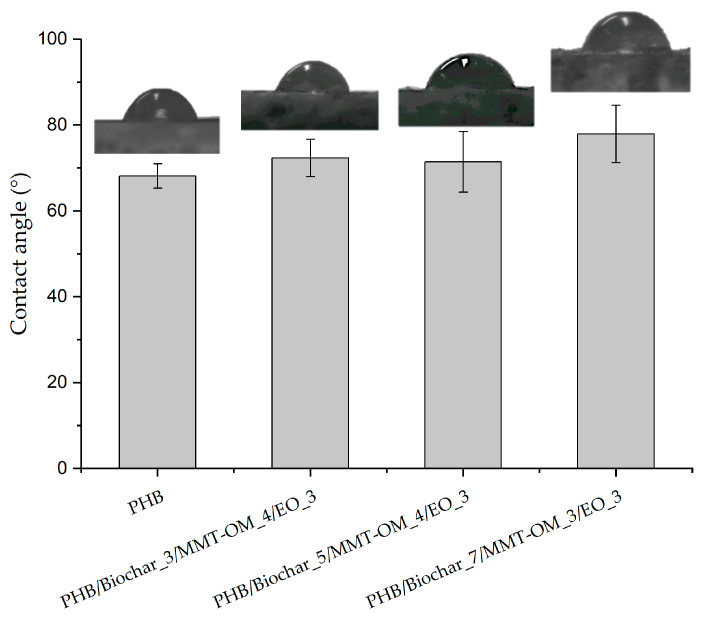
Variation of WCA in the PHB and bionanocomposites.

In order to understand the surface behavior of the different bionanocomposites and PHB, the water contact angle (WCA) was evaluated. As shown in Figure 4, the surface of the different bionanocomposites and PHB have a hydrophilic character, but the bionanocomposites showed an angle greater than 71°, unlike PHB which exhibited an angle of 68°. These results indicate that the presence of biochar, clay, and EO tends to produce a surface with a tendency toward hydrophobicity. This can be observed in the PHB/biochar_7/MMT-OM_3/EO_3 bionanocomposite, which has the highest concentration of biochar. The biochar confers higher hydrophobicity due to its nature as a highly porous material with different pore sizes, as observed in the SEM microphotographs. It has been found that the wetting properties of biochar depend on the pore width and the surface chemistry of the pore walls. For a biochar to be hydrophobic, the pore surface must be hydrophobic, creating a negative capillary pressure that repels water from the pores [56]. It has also been determined that, due to its hydrophobicity, it has high compatibility with different polymers [57].

**Figure 5:**
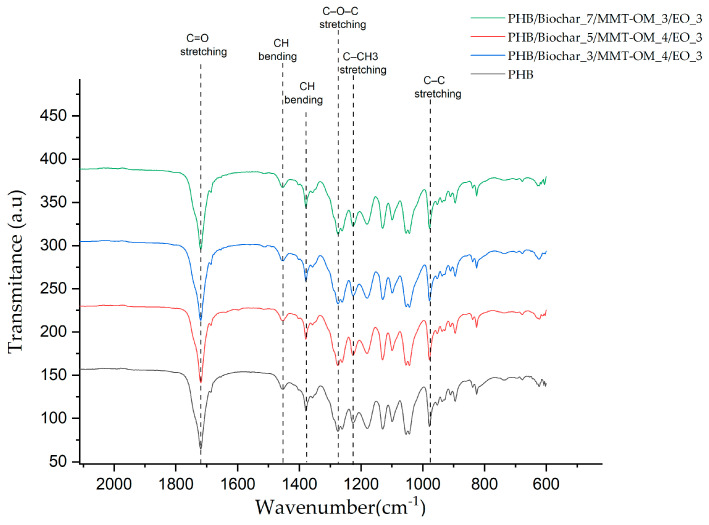
FTIR-ATR spectra for the PHB and bionanocomposites.

An FTIR-ATR assay was performed to determine the possible interactions between the different components of the bionanocomposites. Figure 5 shows the different spectra of the bionanocomposites and PHB. It can be seen that the different spectra show the characteristic absorption bands of PHB, highlighting, among them, the band at 1717 cm^−1^ associated with C=O stretching, the band at 1452 cm^−1^ associated with asymmetric bending of -CH_2_ or -CH_3_, the band at 1377 cm^−1^ corresponding to a symmetrically bending -CH_3_ group, the band at 1276 cm^−1^ corresponding to C-O-C stretching, the band at 1223 cm^−1^ associated with C-CH_3_ stretching, and the band at 980 cm^−1^ corresponding to C-C stretching [58,59,60]. The most significant difference between the bionanocompounds and PHB is a small band appearing at 1511 cm^−1^, which coincides with a characteristic band of eugenol associated with the C=C stretching of the eugenol aromatic ring [61]. The low variability in intensity and position of the peaks of the bionanocomposite is attributed to the lack of formation of new covalent bonds, so that there is only physical interaction occurring between the different components of the bionanocomposites. Similar conclusions were reached by Kumari et al. [62], who obtained PHB/grapeseed oil/MgO nanoparticle composites. It should also be considered that FTIR-ATR is a surface technique and PHB is the major compound (matrix) of the bionanocomposites.

**Figure 6:**
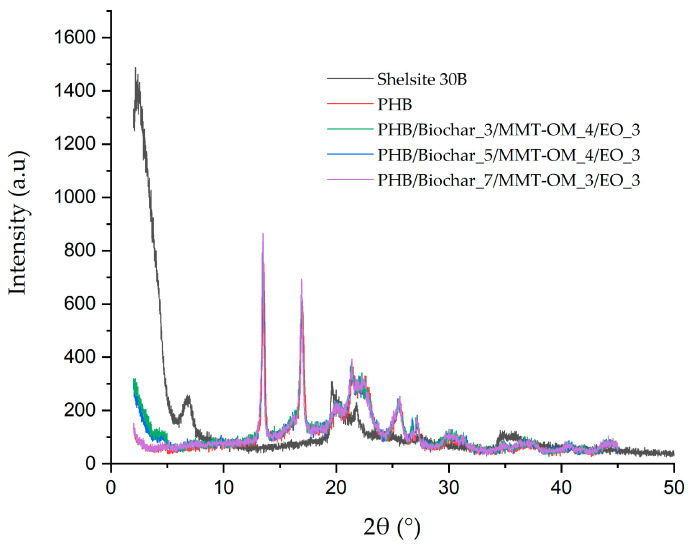
XRD patterns of PHB and bionanocomposites.

X-ray diffraction is one of the techniques used for the detection of the production of bionanocomposites. The lamellar structure of clays renders them hydrophilic by nature. However, they are modified to alter this property, becoming hydrophobic and increasing the spacing between their lamellae. This modification facilitates the enhanced dispersion of the polymer [63]. The bionanocomposite samples were obtained through the use of montmorillonite clay that had been modified with methyl tallow bis-2-hydroxyethyl quaternary ammonium chloride [64]. This modification resulted in a characteristic peak at 2.38°, which corresponds to an interlaminar distance of 3.70 mm, as determined by the Bragg equation (λ = 2d sinθ). Figure 6 indicates that bionanocomposites with a 4% *w*/*w* clay concentration demonstrate displacement at a small angle. This finding suggests that the clay peak is displaced at small angles which fall outside the analyzed range. This observation could be indicative of an intercalated morphology in bionanocomposites with this clay concentration. Such a morphology would permit the polymer and other components that may interact with the structure of the clay modifier to enter. In the case of the other bionanocomposites, the clay peak was not observed, suggesting that these materials exhibit exfoliated morphology. It is important to note that, in these types of materials, it is very difficult for them to present only one type of morphology; they commonly tend to have a combined intercalated–exfoliated morphology. Similar results have been determined by Zhu et al. [65], who obtained polyaniline–montmorillonite-clay nanocomposites, and Garrido-Miranda et al. [38], who obtained (PHB)-thermoplastic starch (TPS)/montmorillonite clay (OMMT)/eugenol.

On the other hand, Figure 6 shows that the bionanocomposites present the characteristic peaks of PHB, i.e., 13.4° (020), 16.8° (110), and 19.9° (021) [66,67], with their respective Miller indices (crystallographic planes). The variation in the intensity of these peaks is indicative of the alteration to the crystal structure of PHB, a consequence of the formation of hydrogen bonds between the compounds [68,69]. For instance, the formation of hydrogen bonds can occur between PHB and essential oils or between PHB and the clay modifier, as these possess hydroxyl groups. In addition, the PHB/biochar_7/MMT-OM_3/EO_3 bionanocomposite showed the highest intensity in the 020 and 010 crystallographic planes.

**Figure 7:**
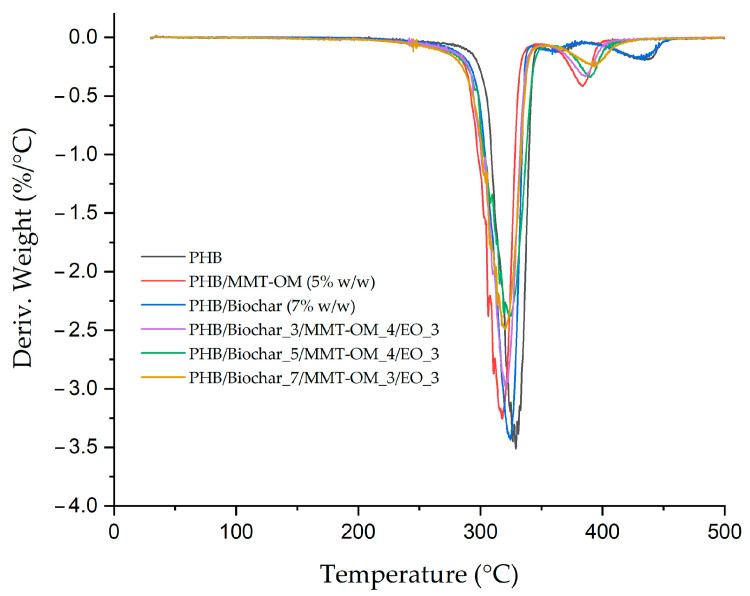
DTGA curve for PHB, PHB–clay blend, PHB–biochar blend, and bionanocomposites.

To determine the impact of the components and their respective concentrations on the thermal characteristics of the bionanocomposite in comparison to PHB, differential scanning calorimetry (DSC) and thermogravimetric analysis (TGA) were employed. As shown in Figure 7, the TGA derivative of various samples was examined, and Table 2 provides a comprehensive overview of the Td, T_5%_, T_10%_, and T_50%_ values. With respect to T_5%_, it is 17 °C less than that of PHB, a phenomenon that can be attributed to the loss of the essential oil mixture Tepa:eugenol (EO) present in the bionanocomposites, specifically the portion not retained in the clay layers or in the biochar pores. The degradation of PHB is evident in the DTGA graph, as shown in Figure 7. The initial decomposition stage (Td_1_) is observed at a temperature of 316 °C and is predominantly attributed to the decomposition of the PHB itself. The second stage (Td_2_) occurs at a higher temperature of 433 °C and is attributed to the decomposition of the commercial PHB additive [51,66]. It is observed that, within the diverse bionanocomposites, there is no substantial variation in the values associated with Td_1_, as it only increases by 4 °C. This suggests that the new material, comprising a blend of PHB, clay, biochar, and essential oils, does not exhibit a significant variation in the Td_1_ of PHB. Consequently, the thermal stability of the bionanocomposite can be ranked as follows: PHB/biochar_5/MMT-OM_4/EO_3 > PHB/biochar_3/MMT-OM_4/EO_3 = PHB/biochar_7/MMT-OM_3/EO_3 > PHB. Researchers such as Alghyamah et al. [70] have obtained biochar/polypropylene composites with 20% *w*/*w* biochar which showed an increase in Td at 30 °C compared to PP. These authors found that the improvement in Td was due to the ability of biochar to scavenge free radicals which delayed the degradation of PP. On the other hand, Musiol et al. [71] obtained poly(lactic acid)/P(3HB-co-4HB) composites with 30% biochar and concluded that the Td of P(3HB-co-4HB) showed no variation and that that of PLA decreased. This decrease was related to the amount and dispersion of biochar, in addition to its effect on the crystallization of PLA molecular chains and PLA–biochar molecular chain interactions. Therefore, the biochar did not have a significant effect on the bionanocomposites, probably because it is present at low concentrations.

**Figure 8:**
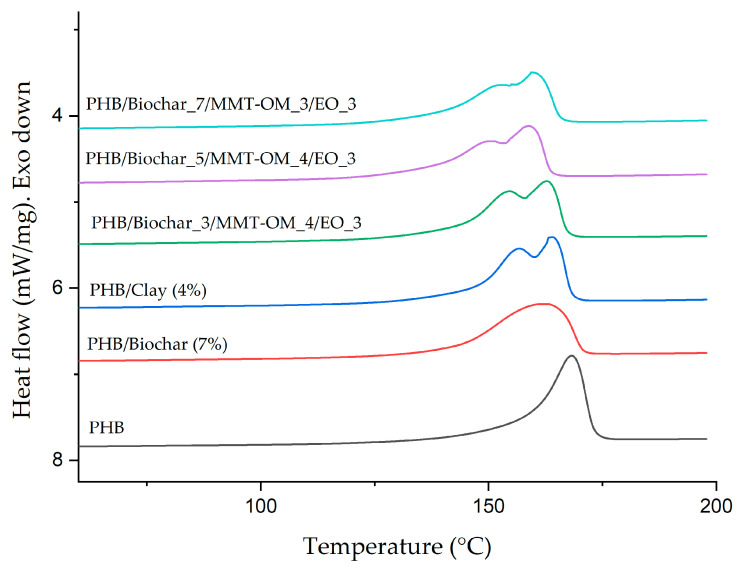
DSC curves obtained for PHB, PHB–clay blend, PHB–biochar blend, and bionanocomposites.

To investigate the thermal analysis in more detail, and to determine the different thermal transitions of the bionanocomposites and PHB, a differential scanning calorimetry (DSC) analysis was performed. The crystallization temperature (Tc), melting temperature (Tm), their respective enthalpies, and the degree of crystallinity of each material were determined and the values obtained are summarized in Table 2. Figure 8 shows the thermograms associated with the second DSC heating of PHB, PHB–clay, PHB–biochar, and bionanocomposites. As shown in Figure 8, PHB shows an endothermic peak at 168 °C and an enthalpy of 73 J/g, which changes when PHB–biochar and PHB–clay composites are obtained. In the first case, the peak shifts to lower temperatures (161 °C) and the enthalpy decreases to 71 J/g, while the composite with clay, when going through the melting–recrystallization–melting process, produces a double endothermic melting peak (Tm_1_ of 156 °C and Tm_2_ of 164 °C). This phenomenon is related to the fact that the clay plates have a nucleating effect that promotes heterogeneous nucleation, generating different crystallite sizes in the melting–crystallization process. M. Jesús Fernández et al. [72] also observed a double peak for their polylactic acid (PLA)/organovermiculite nanocomposites, concluding that organovermiculite acts as a nucleating agent and that, at higher concentrations, Tm_1_ decreases. Garrido-Miranda et al. [51] observed the same phenomenon with PHB–thermoplastic starch/clay bionanocomposites. They determined that the double peak occurs because the clay lamellae hinder recrystallization and cause a reduction in PHB crystal size.

With regard to the presence of biochar in the bionanocomposites, it is more clearly observable in the PHB–biochar blend, as it produces a broader peak with a shift to lower melting (161 °C) and crystallization temperatures (110 °C). The predominant impact of biochar on the crystallization temperature has been documented by Srihanam et al. [73] in their poly(L-lactide)-b-poly(ethylene glycol)-b-poly(L-lactide) (PLLA-PEG-PLLA)/biochar composite. They reported a decrease in Tc of 4 °C for the composite with 5% biochar and attributed these results to the nucleating effect produced by the biochar. Similar results were reported by Liu et al. [54] with respect to their polyethylene glycol/biochar composite, with the decrease in Tc attributed to the fact that the biochar, due to its microstructure, limits the crystallization process.

The bionanocomposites exhibit a double endothermic peak, indicative of the presence of clay, accompanied by a reduced melting temperature. Among these, the PHB/biochar_5/MMT-OM_5/EO_3 bionanocomposite emerges as a notable exception, with a Tm_1_ of 149 °C and a Tm_2_ of 158 °C. This bionanocomposite also exhibits the lowest crystallization temperature, a phenomenon that can be attributed to the presence of biochar, which decreases by 4% compared to PHB. This observation is further substantiated by the degree of crystallinity of this bionanocomposite which was the lowest recorded at 48%. In contrast, the PHB/biochar_3/MMT-OM_4/EO_3 bionanocomposite exhibited the highest degree of crystallinity (53%).

The effect of biochar concentration on the mechanical properties of the bionanocomposites was analyzed by nanoindentation. The elastic modulus and hardness of the different materials were determined and are summarized in Table 3. It is demonstrated that the elastic modulus of the bionanocomposites with 3 and 5% by weight of biochar decreased by 30% and 11%, respectively, in comparison to that of PHB. The PHB/biochar_7/MMT-OM_3/EO_3 bionanocomposite exhibits an elastic modulus of 2.40 GPa. The findings of this study suggest that a concentration greater than 7% results in a material with a higher degree of rigidity compared to PHB. Conversely, lower concentrations yield a material with a greater degree of flexibility compared to PHB. These observations are crucial in determining the ultimate application of the material based on its mechanical properties. For example, a material with high flexibility and resistance to elongation could be suitable for flexible packaging for fruit storage, where adaptability is required without compromising the integrity of the contents. On the other hand, a material with high rigidity and resistance to deformation would be more appropriate for structural packaging designed for food transportation that needs greater protection against impact or mechanical loads.

The hardness results of the bionanocomposites are lower than those of the PHB, indicating that the structure of the bionanocomposites decreases the load resistance. This is indicative of low dispersion or agglomeration of the clay or biochar, a phenomenon which can be observed in the images of the cross-sections (Figure 2). Of the bionanocomposites, PHB/biochar_7/MMT-OM_3/EO_3 stands out as having a hardness similar to that of PHB.

As indicated by the crystallinity results presented in Table 2, it was to be expected that the PHB/biochar_3/MMT-OM_4/EO_3 bionanocomposite would demonstrate optimal mechanical properties, given that PHB is a semicrystalline polymer. However, the obtained results indicate that the PHB/biochar_7/MMT-OM_3/EO_3 bionanocomposite exhibits the most advantageous mechanical properties. Yanji Zhu et al. [74] obtained similar results with respect to their PVA/graphene oxide nanocomposites, concluding that, given the absence of a correlation between the degree of crystallinity and mechanical properties, enhancements in the bionanocomposite would be attributable to charge dispersion and the interaction between the polymer and the nanocomposite charges.

**Figure 9:**
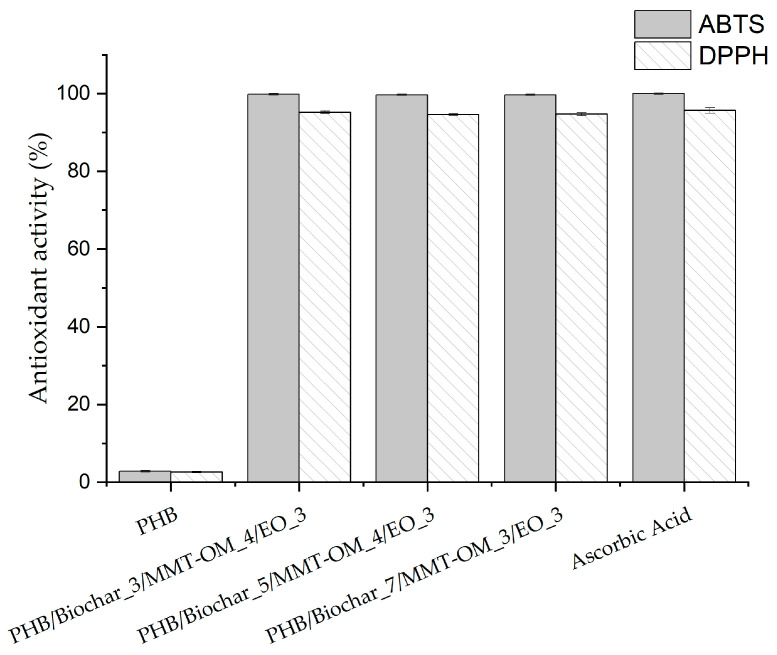
Antioxidant activity (ABTS and DPPH scavenging assay) of the of PHB and bionanocomposites.

An antioxidant active packaging is a promising type of packaging in the area of fatty foods, as the presence of antioxidants is able to retard lipid oxidation and prevent the rancidity process [75]. The bionanocomposites obtained in this study contain a mixture of essential oils of Tepa-eugenol in a 70:30 ratio and a concentration of 3% by weight. The antioxidant activity of these bionanocomposites was analyzed by performing two types of DPPH and ABTS assays, as shown in Figure 9. The PHB was utilized as a control due to it not exhibiting antioxidant activity. Ascorbic acid was employed as a positive control, attaining 100% activity in the ABTS assay and 95% in the DPPH assay. While all the bionanocomposites exhibit high antioxidant activity, reaching values close to 99% in the ABTS test and 94% in the DPPH test, PHB/biochar_7/MMT-OM_3/EO_3 distinguishes itself because of its enhanced physicochemical properties. This demonstrates the elevated antioxidant capacity of the bionanocomposites fabricated with low concentrations (3% *w*/*w*) of Tepa-eugenol (70:30) essential oil, with their antioxidant capacity approaching positive control values. Furthermore, it was determined that the oil mixture demonstrates resilience during the film casting process, resulting in the formation of the bionanocomposites. Moreover, the biochar concentration was found to be insignificant with regard to the antioxidant activity of the material.

The results of the present study are congruent with the findings of previous research conducted by other authors. In their study, Elena Orlo et al. [76] utilized two vinyl resins containing eugenol to coat flexible aluminum foils. They determined that a 5% concentration of eugenol in their materials achieved DPPH radical scavenging values of 81 and 65%, respectively, as well as ABTS radical scavenging values of 1.37 and 6.63%, respectively. On the other hand, Magdalena Woźniak et al. [77] determined that chitosan films with 1% eugenol achieved an antiradical activity (DPPH) of 44%. The notable antioxidant properties of eugenol are attributed to its capacity to dimerize, thereby forming dehydrodieugenol [78], such a process facilitating the stabilization of another radical molecule [38].

## 4. Conclusions

The different properties of PHB bionanocomposites containing different combinations of biochar, clay (MMT-OM), and a constant concentration of the essential oil mixture (Tepa:eugenol) (EO) were determined. The novel material exhibited a porous surface, as evidenced by scanning electron microscopy (SEM), and the cross-section revealed cavities concomitant with the presence of biochar. Furthermore, the XRD analysis revealed the disappearance of the peak associated with clay, suggesting that the bionanocomposites possessed an exfoliated–intercalated morphology. The bionanocomposite with 7% by weight of biochar exhibited the highest contact angle with water, close to 71°, and the highest elastic modulus (2.40 GPa). Analysis of the thermal properties of the bionanocomposites revealed minimal differences with respect to PHB, the most significant being the melting temperature, which decreased by 11%. The crystallization temperature and decomposition temperature did not show significant variations. Finally, the analysis of the antioxidant properties against DPPH and ABTS demonstrated values of 94% and 95% of antioxidant activity, respectively. These values are comparable to those of the positive control. In summary, the present material, composed of a PHB/biochar/clay/Tepa:eugenol bionanocomposite, substantiates its status as a novel material for application in the food packaging industry, particularly for foods that are susceptible to the oxidation process. The subsequent step for this material is to analyze its ability to increase the shelf life of food that is prone to oxidation, such as bananas. Additionally, a biodegradability evaluation will be conducted under composting conditions to confirm its status as an active biodegradable material.

## Figures and Tables

**Table 1:** Name and percentage by weight of each sample.

Sample	Biochar (% *w*/*w*)	MMT-OM (% *w*/*w*)	EO (% *w*/*w*)
PHB	0	0	0
PHB/biochar_3/MMT-OM_4/EO_3	3	4	3
PHB/biochar_5/MMT-OM_4/EO_3	5	4	3
PHB/biochar_7/MMT-OM_3/EO_3	7	3	3

**Table 2:** Thermal properties obtained from TGA and DSC heating curves for PHB and bionanocomposite films.

Sample	Tm_1_ (°C)	Tm_2_ (°C)	Tc (°C)	ΔHm (J/g)	ΔHc (J/g)	Xc (%)	Td (°C)	T_5%_ (°C)	T_10%_ (°C)	T_50%_ (°C)
PHB	168.3	-	112.9	72.70	63.03	49.79	316.2	289.4	300.5	315.5
PHB/biochar_3/MMT-OM_4/EO_3	154.8	162.8	110.8	70.13	63.13	53.37	320.6	277.6	296.8	319.7
PHB/biochar_5/MMT-OM_4/EO_3	150.3	158.7	108.8	62.65	60.49	48.76	324.9	275.1	296.5	324.4
PHB/biochar_7/MMT-OM_3/EO_3	152.4	159.5	109.7	63.91	58.41	50.32	320.1	272.2	293.0	320.0

**Table 3:** Values of elastic modulus and hardness of PHB and bionanocomposites.

Sample	Elastic Modulus (GPa)	Hardness (MPa)
PHB	2.08 ± 0.23	134.05 ± 12.68
PHB/biochar_3/MMT-OM_4/EO_3	1.86 ± 0.19	85.56 ± 5.19
PHB/biochar_5/MMT-OM_4/EO_3	1.47 ± 0.16	66.80 ± 6.02
PHB/biochar_7/MMT-OM_3/EO_3	2.40 ± 0.96	128.96 ± 30.78

## Data Availability

The original contributions presented in this study are included in the article. Further inquiries can be directed to the corresponding author.

## Abbreviations

The following abbreviations are used in this manuscript:

MMT-OM	Organic montmorillonite Shelsite 30B
EO	Tepa:eugenol (70:30) essential oil mixture
DPPH	2,2-diphenyl-1-picrylhydrazyl radical
ABTS	2,2′ azino-bis (3-ethylbenzothiazoline)-6-sulfonic acid
PHB	Polyhydroxybutyrate
Td	Degradation temperature
Tm	Melting temperature
Tc	Crystallization temperature
ΔHm	Enthalpies of fusion
ΔHc	Enthalpies of crystallization
Xc	Degree of crystallinity
